# Supplementary material for: Effects of Fermented Bamboo Shoot Processing Waste on Growth Performance, Serum Parameters, and Gut Microbiota of Weaned Piglets
Source: Animals (Basel). 2022 Oct 11;12(20):2728. doi: 10.3390/ani12202728 (PMC9597720; doi:10.3390/ani12202728)

Table S1. Serum biochemical indices of weaned piglets fed different feeds containing different fermented bamboo shoot processing wastes (FBSPW). Control, A, B, and C were the piglets fed basal diet, basal diet with 4% FBSPW, basal diet with 8% FBSPW, and basal diet with 12% FBSPW, respectively.

Items	A	B	C	CK
GLU	7.46±1.75	7.6±1.41	7.04±0.95	7.71±0.83
T-cho	2.23±0.40	1.84±0.14	2.26±0.30	2.29±0.21
TG	0.34±0.02^{b}	0.36±0.02^{b}	0.39±0.08^{b}	0.58±0.07^{a}
LDL-C	0.55±0.14	0.58±0.06	0.58±0.10	0.61±0.19
TP	43.02±1.71	52.57±10.27	45.65±6.66	47.82±0.24
ALB	27.60±3.16^{ab}	30.31±4.67^{a}	27.18±2.29^{ab}	21.24±3.32^{b}
HDL-C	2.01±0.17^{ab}	2.16±0.18^{a}	1.9±0.32^{ab}	1.59±0.16^{b}
BUN	1.74±0.46^{ab}	1.35±0.17^{b}	1.24±0.06^{b}	2.81±0.69^{a}

Figure S1. Alpha diversity indices of colon and cecum microbiota of weaned piglets fed different feeds containing different fermented bamboo shoot processing wastes (FBSPW). (A), feature number; (B) Shannon index; (C) Simpson index; (D) ACE index; (E) PD index; and (F) Goods' coverage. Control, A, B, and C were the piglets fed basal diet, basal diet with 4% FBSPW, basal diet with 8% FBSPW, and basal diet with 12% FBSPW, respectively.

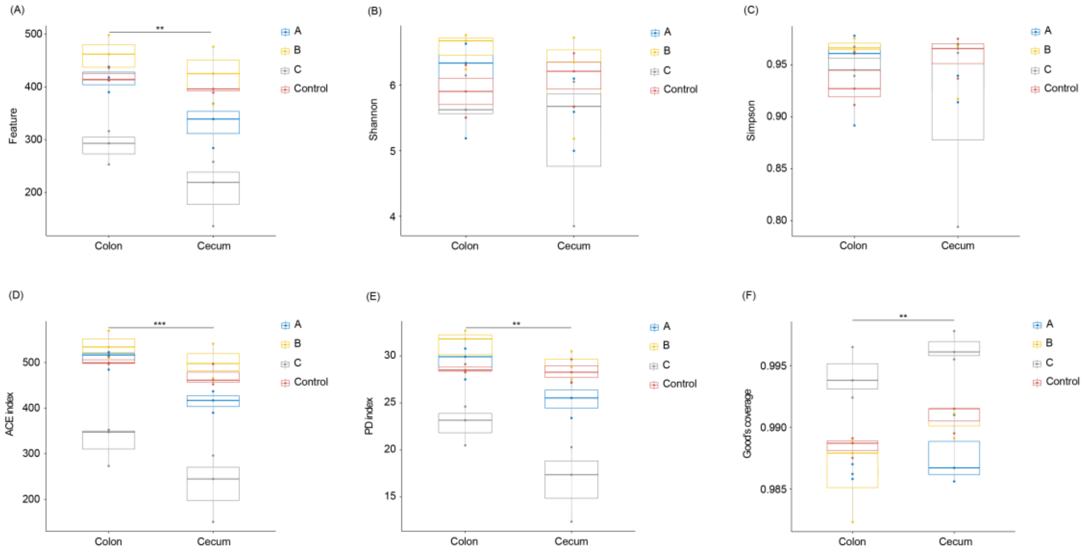

Figure S2. Relative abundance changes of gut microorganisms of weaned piglets fed different feeds containing different fermented bamboo shoot processing wastes (FBSPW). Control, A, B, and C were the piglets fed basal diet, basal diet with 4% FBSPW, basal diet with 8% FBSPW, and basal diet with 12% FBSPW, respectively.

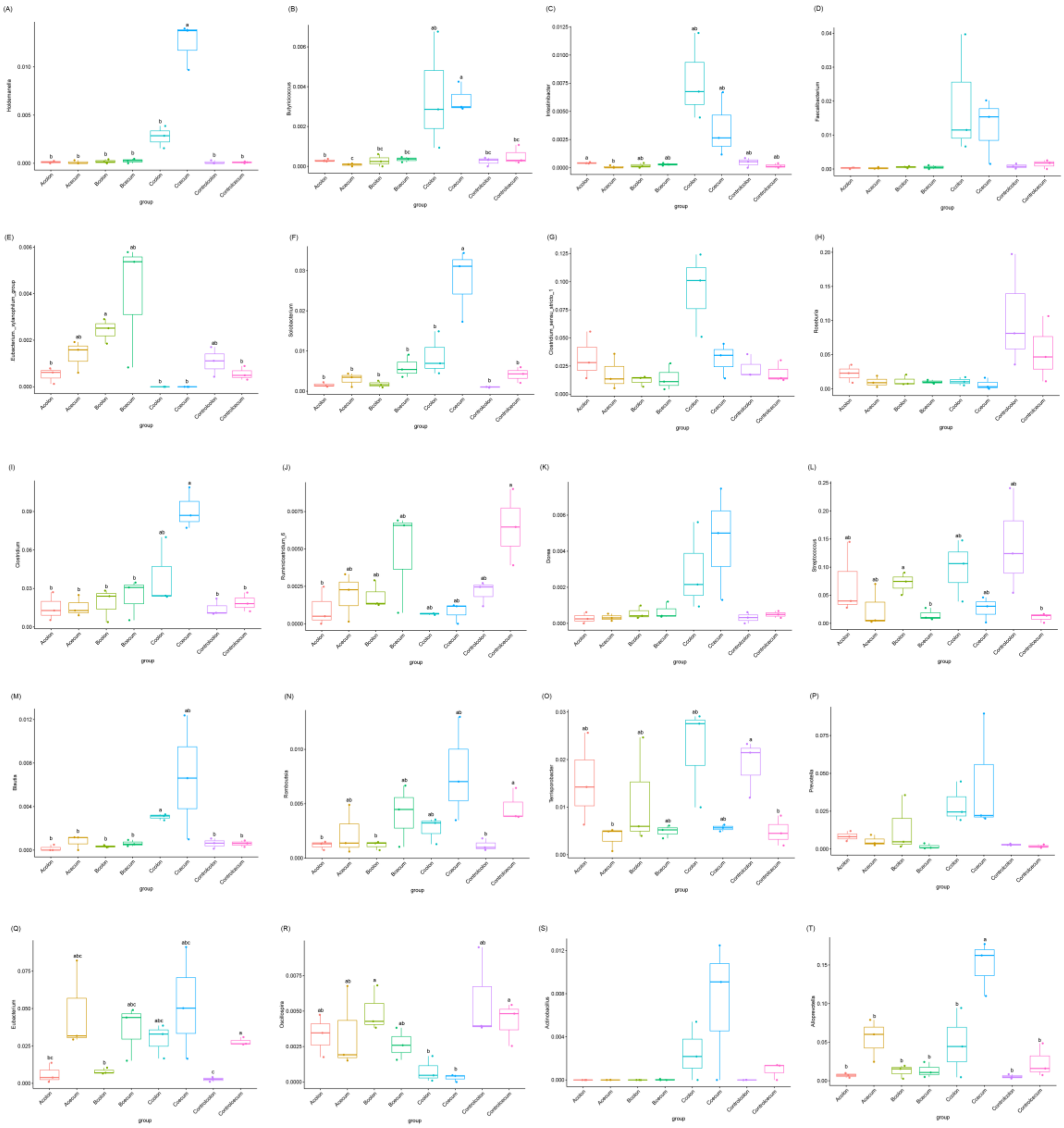

Supplement: Supplementary file 1 [file animals-12-02728-s001.zip › animals-1840129-supplementary.pdf]
